# Leg Length Discrepancy After Total Hip Arthroplasty: A Review of Clinical Assessments, Imaging Diagnostics, and Medico-Legal Implications

**DOI:** 10.3390/healthcare13121358

**Published:** 2025-06-06

**Authors:** Luca Bianco Prevot, Livio Pietro Tronconi, Vittorio Bolcato, Riccardo Accetta, Lucio Di Mauro, Giuseppe Basile

**Affiliations:** 1 IRCCS Ospedale Galeazzi–S. Ambrogio, 20157 Milan, Italy; riccacc@gmail.com; 2 Residency Program in Orthopaedics and Traumatology, University of Milan, 20122 Milan, Italy; 3 Department of Human Science, European University of Rome, 00163 Rome, Italy; liviopietro.tronconi@unier.it; 4 Maria Cecilia Hospital, GVM Care & Research, 48033 Cotignola, Italy; 5 Maria Beatrice Hospital, GVM Care & Research, 50121 Firenze, Italy; vbolcato@gvmnet.it; 6 Department of Legal Medicine, University of Catania, 95124 Catania, Italy; dr.luciodimauro@gmail.com; 7 Department of Biomedical Sciences and Public Health, University “Politecnica delle Marche” of Ancona, 60124 Ancona, Italy; basiletraumaforense@gmail.com

**Keywords:** leg length discrepancy, total hip arthroplasty, medico-legal implications

## Abstract

Background/Objectives: Total hip arthroplasty (THA) is a widely performed procedure to alleviate pain and improve function in patients with hip disorders. However, leg length discrepancy (LLD) remains a prevalent complication. LLD can cause gait disturbances, back pain, postural imbalance, and patient dissatisfaction, along with significant medico-legal implications. This review examines the evaluation, management, and medico-legal aspects of LLD. Methods: The review analyzed literature on the prevalence, evaluation methods, and management strategies for LLD in THA. Radiographic and clinical assessment tools were considered, alongside factors such as pelvic obliquity and pre-existing conditions. The importance of preoperative planning, intraoperative techniques (including computer-assisted methods), and comprehensive documentation was evaluated to address both clinical and legal challenges. Results: The review shows that leg length discrepancy (LLD) following total hip arthroplasty (THA) occurs in 3% to 30% of cases, with mean values ranging from 3 to 17 mm. LLD may result from anatomical or procedural factors, and effective evaluation requires both radiographic imaging and clinical assessment. Preoperative planning plays a critical role in accurately assessing anatomical parameters and selecting appropriate prosthetic components to preserve or restore limb length symmetry. Advanced intraoperative techniques, including computer-assisted surgery, help reduce LLD incidence. While some complications may be unavoidable, proper documentation and patient communication, particularly regarding informed consent, are essential to mitigate medico-legal risks Conclusions: LLD after THA requires a multidimensional approach incorporating clinical, radiological, biomechanical, and legal considerations. Effective preoperative and intraoperative strategies, combined with robust communication and documentation, are essential to minimize LLD and its associated risks. A focus on precision and patient-centered care can improve outcomes and reduce litigation.

## 1. Introduction

Total hip arthroplasty (THA) is one of the most common and well-established orthopedic procedures, primarily aimed at improving the quality of life for patients suffering from degenerative, traumatic, or inflammatory conditions of the hip joint [1]. Despite advancements in surgical techniques and the materials used, the risk of post-operative complications persists [2].

Among these, leg length discrepancy (LLD) is one of the most frequent and clinically significant issues, with a prevalence ranging from 3% to 30% according to the literature [3,4].

The review provides a wide range of results on LLD, within which other differences are relevant, such as the nature of the length discrepancy and the need for shoe modifications. These discrepancies in the data may indicate differences in surgical planning and techniques, patient demographics and selection for surgery, and assessment methods. This wide variability suggests that research in this area is inconsistent and that further studies are needed to provide more accurate and comparable data. More recent studies have reported a modest but consistent reduction—by a few percentage points—in the incidence of post-operative discrepancies, demonstrating how technological advances in hip prosthesis implantation have led to more precise outcomes, particularly in restoring limb length accuracy [5].

Moreover, this includes both anatomical and functional discrepancies, which are prevalent and more complex to describe. Part of the purpose of this qualitative review given the medico-legal slant is to initiate a discussion on a complex topic on which a critical synthesis is lacking. LLD can range from minimal and clinically irrelevant to significant, leading to adverse outcomes in both biological functions and the patient’s quality of life. Functionally, it may cause postural imbalances, chronic low back pain, gait difficulties, and increased wear on prosthetic components, with a direct impact on the patient’s quality of life [6].

Beyond its physical effects, LLD also determines medico-legal implications, as it is one of the leading alleged causes of malpractice litigation in prosthetic surgery [7]. From a medico-legal perspective, LLD raises concerns regarding medical liability: it is essential to distinguish between an unavoidable complication, inherent to the complexity of the procedure and patient characteristics, and an avoidable error, resulting from a surgical team error in the peri-operative and rehabilitative process [8]. Furthermore, medico-legal evaluation of LLD requires at first the assessment of the causal link between the surgery and the adverse outcome, then the application of standardized criteria for surgeons’ conduct assessment compared to best evidence, and finally the assessment and evaluation of the damage.

The aim of this review is to provide a comprehensive definition of LLD, the methods that can be used to assess limb length, and the overall medico-legal evaluation of this type of complication. Some recommendations for clinical risk management applications are provided to strengthen the practical implications of medico-legal considerations.

## 2. Anatomical and Functional Lower Limb Discrepancy: Definition and Assessment

**Figure 1:**
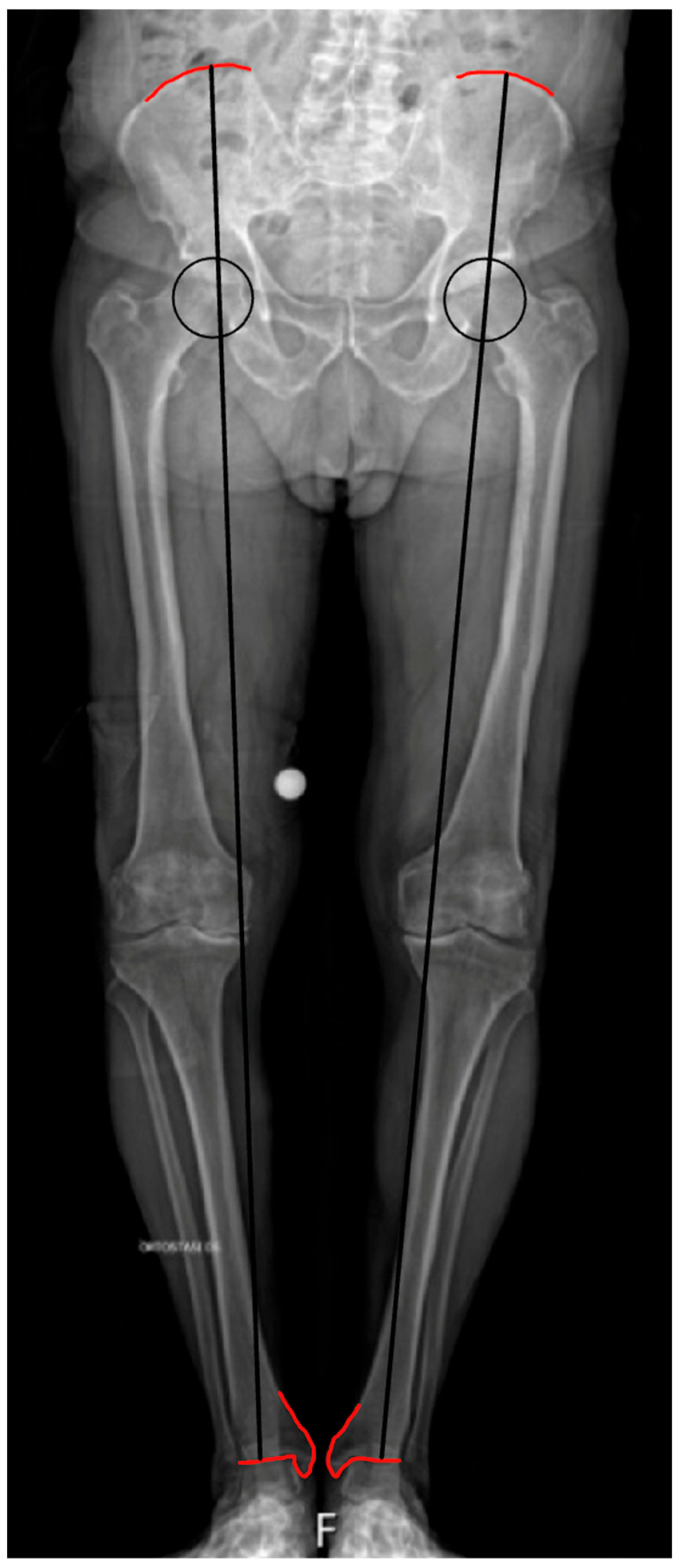
Illustration of lower limb length measurement on a full-length radiograph of both legs: a line connects the highest point of the iliac crest (red lines) to the midpoint of the tibio-tarsal joint line (red lines), passing through the femoral head’s center of rotation.

Anatomical LLD is a true discrepancy resulting from a difference in the length of the leg. It is possible to define mild (less than 10 mm), moderate (between 10 and 20 mm), and severe (more than 20 mm) LLD [9]. There are several methods that allow the evaluation of LLD. In teleo-roentgenogram imaging, LLD is calculated by measuring the difference in the length of line segments drawn from the top of the femoral head to the center of the ankle on both sides of the body Adjustments must be made for hip or knee flexion contractures. Another critical factor is the rotation center height of femoral heads, as variations here may cause LLD despite similar limb lengths. Proper alignment during imaging ensures consistent measurements (Figure 1).

**Figure 2:**
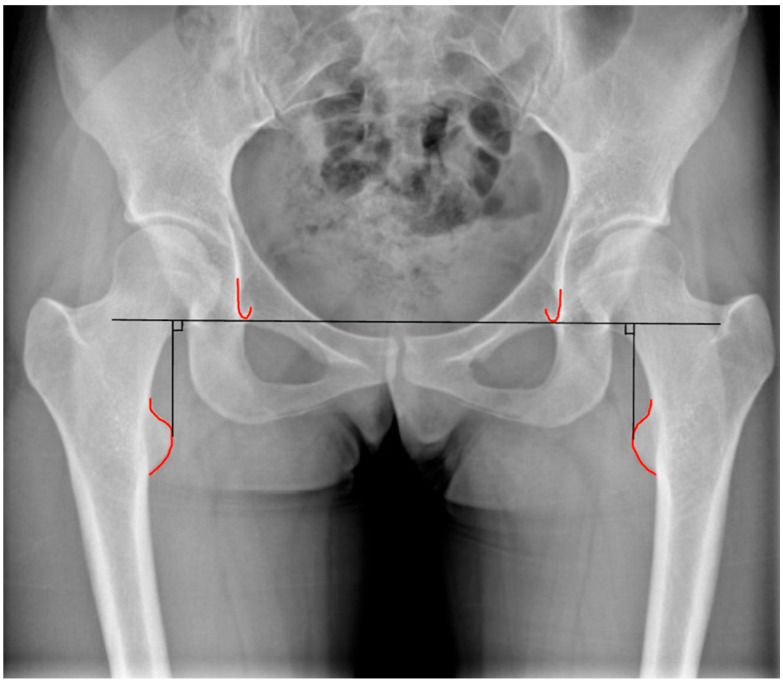
Assessment of discrepancies in lower limb length related to the hip can utilize different reference points: the radiological teardrop.

**Figure 3:**
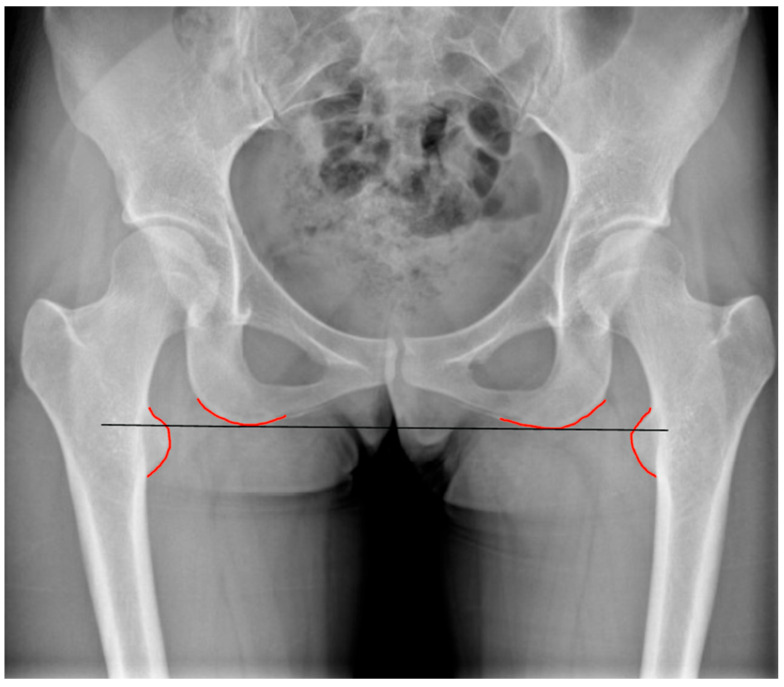
Assessment of discrepancies in lower limb length related to the hip can utilize different reference points: the bi-ischial line.

An evaluation of LLD can also be performed through pelvic radiographs, which are simpler to obtain and commonly used in preoperative assessments for hip arthroplasty. Radiographic LLD assessment utilizes pelvic landmarks, including the ischial tuberosity (IT), the bi-iliac line (BI), and the linear passing through the teardrops as references. A perpendicular line is drawn through femoral markers, such as the lesser trochanter (LT) [10,11]. The measured distances (IT-LT, BI-LT) on both sides are compared to determine LLD (Figure 2 and Figure 3).

In the postoperative evaluation, specific landmarks are identified, such as the bi-ischial line or radiological teardrops for the pelvis, along with the centers of the lesser trochanters for the femurs. Measuring the distance between these landmarks before and after total hip arthroplasty (THA) allows for an assessment of the limb length variation caused by the surgical procedure [12].

Functional LLD does not reflect a measure discrepancy of the legs that can be demonstrated through clinical or radiographic evaluation, but rather a sensation reported by the patient. Unlike anatomical or structural LLD, which results from anatomical differences due to bone shortening, fractures, or congenital deformities, functional LLD arises from biomechanical imbalances within the musculoskeletal system.

**Figure 4:**
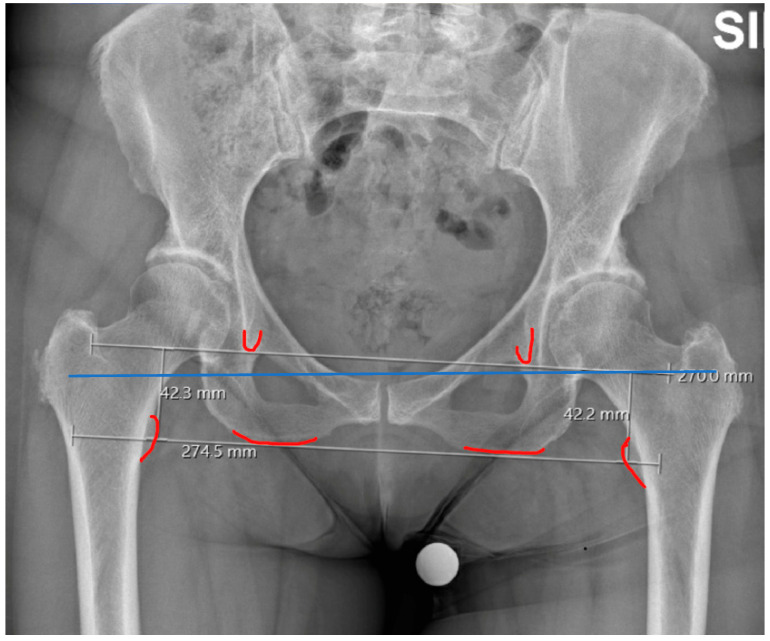
A PO angle is the angle between the horizontal plane (blue lines) and the inter-teardrop line. Although the lower limbs are of equal length, as shown by the line connecting the two radiological teardrops, the pelvis appears oblique, as indicated by the angle between the inter-teardrop line and a line parallel to the horizontal plane.

Factors contributing to functional LLD include pelvic obliquity (PO) caused by abnormalities in the limbs or spine. A balanced distribution of body weight across both hips is essential for maintaining an upright posture. When PO occurs, this balance is disrupted, potentially leading to secondary lumbar scoliosis, rapid spinal degeneration, and abnormal gait patterns. PO can be measured through anteroposterior standard pelvic radiography. A PO angle is the angle between the horizontal plane and the inter-teardrop line which allows for the quantification of pelvic obliquity [13]. Figure 4. Another common contributor to the sensation of LLD is postoperative gluteal muscle tension, especially following procedures like total hip arthroplasty. After surgery, the gluteal muscles may experience increased tension as they adapt to changes in joint alignment and biomechanics. This muscular imbalance can give the impression of leg length discrepancy, even though no structural shortening has occurred. Fortunately, this type of tension typically resolves within six to twelve months as the muscles undergo rehabilitation and neuromuscular adaptation [14,15].

In some cases, spinal factors play a role in the development of functional LLD. Conditions like lumbar scoliosis, herniated discs, or degenerative spinal diseases can cause a tilt in the pelvis, further contributing to the sensation of uneven leg length.

## 3. Lower Limb Discrepancy in THA

The prevalence of lower limb length discrepancy after primary total hip arthroplasty ranges from 3% to 30% [5], with reported variations between 3 mm and 70 mm. Mean discrepancies typically fall within 3 to 17 mm [16]. LLD has been associated with complications such as back pain, sciatica, neuritis, gait disturbances, patient dissatisfaction, dislocation, and early loosening of prosthetic components. In severe cases, LLD can lead to significant morbidity, dissatisfaction, and even revision surgery.

Achieving perfect leg length equalization post-THA remains a challenge. For example, Love et al. found that 18% of patients experienced lengthening exceeding 1.5 cm, with 6% requiring shoe corrections [17]. Similarly, Williamson et al. reported an average LLD of 16 mm, with 27% of patients needing shoe lifts, while Djerf and Wahlstrom observed discrepancies in up to 50% of their cases [18]. Recent studies indicate a significant reduction in the proportion of patients experiencing LLD after THA; notably, Abouelela et al. reported that only 5.3% of patients had a discrepancy exceeding 15 mm [19], while Gallo et al. reported that patients with a discrepancy > 10 mm were 16% [20].

The reduction in leg length discrepancies reported in the literature can be attributed to several factors, including increased awareness among orthopedic surgeons—partly due to the potential medico-legal implications of this complication. Improvements in surgical techniques and intraoperative assessment methods have also played a key role. Furthermore, the advent of technologies such as intraoperative navigation has significantly enhanced the ability to accurately evaluate limb length during surgery, helping to prevent both over- and under-lengthening.

Although discrepancies under 10 mm are typically well tolerated by most patients, even minor disparities can lead to dissatisfaction in some cases. Most often, the operated limb is lengthened rather than shortened. Studies suggest that approximately 50% of cases show an LLD of ≥10 mm [21], with 15–20% of these patients requiring shoe modifications for correction. Importantly, there is a fundamental interplay between leg length and hip stability in THA. Lengthening the operative limb tightens the surrounding soft tissues, enhancing stability, whereas shortening leads to laxity and increases the risk of dislocation. As a result, leg length and stability are two interdependent aspects of the surgical outcome [22].

In a real-world setting, achieving both optimal stability and perfect leg length symmetry is not always feasible. Stability remains the primary objective, and in certain scenarios, the surgeon may need to prioritize it over precise leg length equality. Therefore, it is crucial for both the patient and the surgeon to maintain realistic expectations regarding the procedure, to be properly and comprehensively disclosed during the informed consent process. While absolute symmetry cannot always be guaranteed, stability and limb length should be managed within acceptable limits to ensure functional and satisfactory outcomes.

Another significant issue is the subjective perception of LLD. Approximately one-third of patients report feeling a discrepancy in limb length post-THA [23]. However, studies reveal that only 36% of these cases involve an actual anatomical discrepancy, while the remaining 64% are due to functional LLD [24]. Functional discrepancies may result from factors such as pelvic obliquity, muscular tension, or altered biomechanics following surgery.

However, numerous psychological aspects related in general to post-surgical satisfaction and expectations must be taken into account and are central to the interaction with the patient for the purpose of indication for surgery.

## 4. Preoperative Planning and Intraoperative Assessment

**Figure 5:**
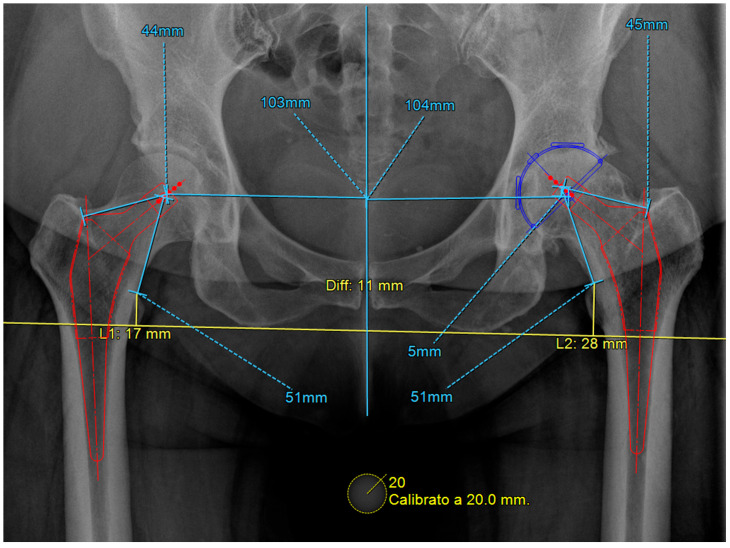
Templating of femoral and acetabular components in anteroposterior view.

Preoperative planning plays a crucial role in achieving consistent and predictable outcomes in contemporary hip arthroplasty (Figure 5).

This process allows the surgeon to mentally prepare for the procedure by carefully analyzing clinical and radiographic data. On the femoral side, templating should focus on optimizing both limb length and femoral offset to enhance the hip joint’s biomechanics [25]. Preoperative templating is often used to guide femoral neck resection and component sizing in total hip arthroplasty even if correct component sizing is achieved in only 60% of cases [26]. Intraoperative methods for managing LLD involve measuring the distance between pelvic and femoral reference points.

The surgical approach should be taken into consideration; in particular, it is important to note that the anterior approach has a lower incidence of LLD greater than 10 mm, with only 1.2% of cases compared to 3.7% for the postero-lateral approach [27]. Traditional techniques often use the greater trochanter as a landmark, supplemented by devices such as iliac pins, calipers, or sutures. The suture-tied technique showed a strong correlation between intraoperative limb length measurements and postoperative limb length, with a correlation coefficient of *r* = 0.86 [28,29]. However, these methods are prone to variability due to changes in hip position, landmark displacement, or inconsistent referencing. Techniques involving the use of Steinmann pins have shown a good correlation with postoperative radiographic assessment of limb length (*r* = 0.84), but they are undoubtedly invasive, as they require the insertion of a pin into the infracotyloid groove of the acetabulum. Computerized navigation has been described, but many are cumbersome, expensive, or have steep learning curves. Errors can also arise from slight variations in femoral abduction/adduction, resulting in discrepancies up to 17 mm [30]. After the implantation of the prosthetic component, limb length can be directly assessed by evaluating the position of the medial malleoli if the patient is in a supine position, while the position of the patellae or heels can be evaluated when the patient is in a lateral decubitus position. Other assessments that can indicate proper limb length and stability include the Shuck test and the drop-kick test [31]. Simpler methods have emerged, such as placing a stable pelvic reference pin and using sutures or other fixed markers to measure and maintain limb length. These techniques hinge on maintaining stable reference points and consistent leg positioning throughout surgery. Recent advancements, including the use of computer navigation, offer greater precision but are hindered by steep learning curves, high costs, and reliance on surgeon-controlled reference points, which can introduce errors [32,33]. Simple techniques, such as the Judd pin and suture method, have shown promise by providing stable pelvic reference points and clear measurement guides, although their success is contingent upon maintaining consistent leg positioning and stable pin placement.

## 5. Clinical Relevance of Lower Limb Discrepancy

Patient dissatisfaction is a frequent issue following THA [34], with leg length discrepancy being cited as one of the leading causes of clinical complaints and litigation. Patients typically notice discrepancies greater than 10 mm [35], which can significantly impact their functional recovery and overall satisfaction. Studies have highlighted links between LLD and conditions such as back pain, lower back pain, or sciatic nerve pain [36], as well as issues like gait abnormalities and joint instability [37]. LLD after THA has been suggested to potentially heighten the risk of developing osteoarthritis in the affected lower limb [38], though this remains a topic of debate. Lengthening of the limb following THA has also been associated with an elevated likelihood of aseptic loosening [39]. In some cases, LLD may necessitate revision surgery due to the high clinical relevance [40]. A significant challenge is determining when a discrepancy in the length can be considered acceptable or not, from a clinical point of view. The threshold separating acceptable from unacceptable LLD has not been definitively established and users’ perceptions must be considered [41]. In fact, studies have reported that most subjects tolerate LLD up to 10 mm, while others claim that even minor discrepancies could lead to dissatisfaction with relevant disability, depending on individual biomechanical, neurological, and psychological factors [42]. Those cases with moderate discrepancies may initially experience discomfort during the first few months, which often subsides over time; however, about 15% of patients remain symptomatic, limiting daily and work activities. This is clinically relevant to understanding how to prevent and better manage LLD, as even anatomical LLDs are associated with a purely functional component. This then has medical-legal issues, in terms, particularly of the risk of litigation after surgery by the patient, and underlines the interest in studying the phenomenon of LLD as a whole, anatomically and functionally, as dissatisfaction remains a central, cross-cutting problem [43].

Conversely, lengthening exceeding 10 mm has been linked to limping, pelvic misalignment, the necessity for shoe lifts, and higher feelings of dissatisfaction [44].

Then, defining the acceptable threshold for LLD remains critical to balancing implant stability and limb length in a single patient and considering its own acceptance threshold [45].

In the preoperative phase, in order to be a candidate for surgery, it is then important also to make a somewhat psychological assessment, taking into account how functional LLD affects the outcome from a clinical -and medico-legal- point of view, such in the cases of other major surgical branches. If it is necessary to select patients and provide them with correct information about the expected clinical outcomes, especially if major depressive symptoms are documented and under treatment -and are reported with higher rates of dissatisfaction.

## 6. Medico-Legal Considerations

Despite technological and surgical advances, post-operative leg length discrepancy continues to be a motivation for litigation, with an impact on the physical and functional harm and medical conduct assessment. The literature indicates that discrepancies greater than 2 cm are most frequently involved in litigation, as they lead to more significant and persistent clinical effect assessment [43].

The key issue is identifying the causes of residual limb length discrepancy and assessing the preventive measures adopted to minimize its occurrence.

Regarding the technical-medico-legal and orthopedic assessment of medical liability, it is essential to distinguish between the following:

*Unavoidable complications*, inherent to the complexity of the surgical procedure managed according to the standard of care.

*Avoidable errors*, arise from surgeon’s errors, such as inadequate preoperative planning, the lack of intraoperative measurement techniques, or improper post-operative load and rehabilitation.

Case law requires that the physician demonstrates having taken all necessary precautions to reduce the risk of foreseeable complications, including the use of validated intraoperative techniques for controlling limb length. The use of intraoperative measurement methods, such as the placement of bone markers or pelvic reference pins, direct comparisons using calipers or suturing tools, or computer navigation technologies, has proven effective in significantly reducing the incidence and magnitude of post-operative LLD [46].

The summary documentation of these actions in the surgical report or in the patient information are therefore elements that support the due and high diligence exercised by the surgeon to control the risk of complications.

In this context, patients must be informed that the primary goal of the procedure is implant stability and functional recovery, specifying that a minimum LLD, and in any case less than 10 mm, may be desired in order to ensure more stability over time and thus not represent an adverse, i.e., negative and undesirable event. They must also understand that another part of the residual discrepancy could be unmanageable and unavoidable and may fall within acceptable standards of care [47].

The management and medico-legal analysis of post-operative discrepancies become more complex in the presence of pre-existing comorbidities such as arthritis, rheumatologic diseases, muscle hypotrophy, or peripheral neuropathies, which may influence both the severity of perceived LLD and the patient’s ability to adapt to a mild or moderate discrepancy. It is essential to determine whether the discrepancy represents a primary cause or an aggravating factor of pre-existing clinical issues. Preoperative documentation must then include a detailed history demonstrating that such conditions were considered and discussed during surgical planning. The anamnesis is so central for the consultant in litigation as it supports the correct surgical indication and allows him to distinguish pre-existing signs and symptoms from supervening ones. The medical history must clearly identify risk factors that may affect limb length measurements after prosthesis implantation, such as the presence of significant pre-existing discrepancies or hip dysplasia.

**Figure 6:**
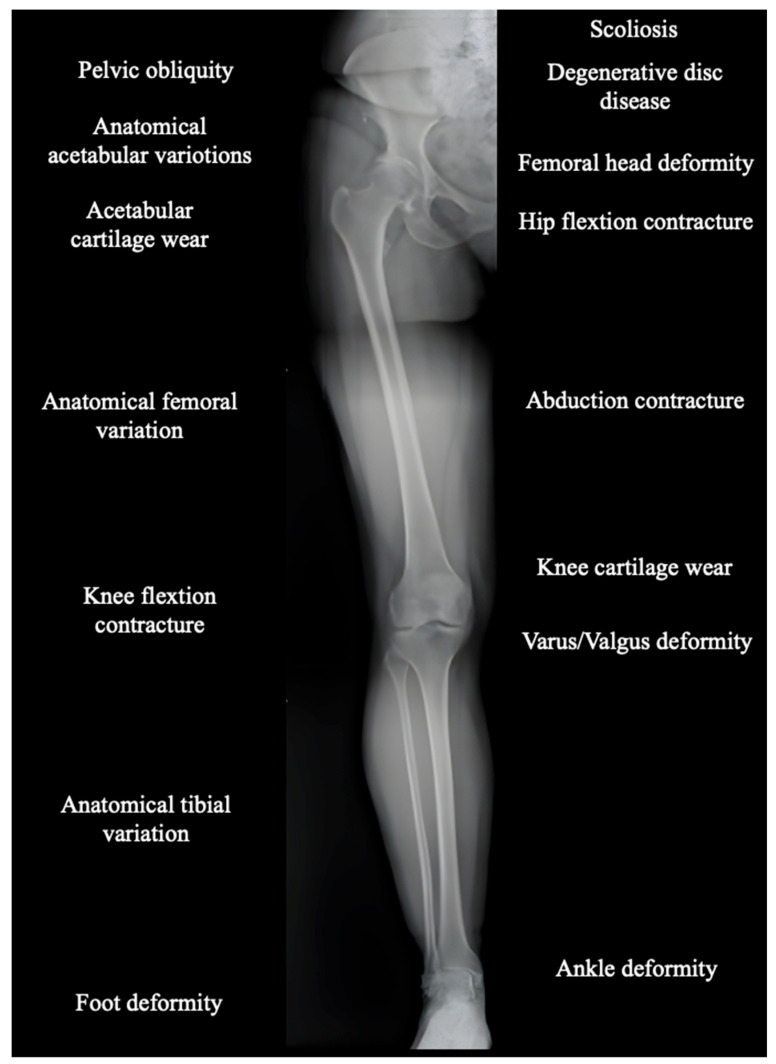
Example of long-limb EOS imaging showcasing the various potential sources contributing to leg length discrepancy.

Functional alterations may arise not only from limb length but also from factors such as axial misalignments (varus/valgus), femoral or tibial rotational issues, and foot or ankle pathologies, often prior to and independent of surgery [46]. (Figure 6).

Another critical factor in assessing LLD is the potential presence of pelvic obliquity, which can significantly affect limb length measurement. Pelvic obliquity may result from pre-existing conditions such as scoliosis or musculoskeletal asymmetries or may be induced postoperatively [45]. Failing to identify and account for this condition can lead to an incorrect assessment of LLD severity and, consequently, suboptimal surgical management. A study on patients with spinal deformities revealed that pelvic obliquity ≥ 3° or a discrepancy ≥ 10 mm was associated with increased risks of post-operative misalignments and complications [48]. To achieve a more comprehensive evaluation, methods combining the patient’s functional posture with radiographic technologies that capture multiple LLD sources, such as scoliosis, anatomical limb variations, and musculoskeletal contractures, are necessary. The surgeon must demonstrate having carefully evaluated and documented the presence of pelvic obliquity, for instance, using standard upright radiographs or preoperative three-dimensional imaging, which provides a more accurate estimate of pelvic alignment and limb length. A simple radiographic evaluation of the pelvis can be limiting, as it only detects discrepancies related to the hip without considering the rest of the lower limb [49].

LLD can originate from alterations in other segments, such as the distal femur, tibia, or even the foot. Then, full-leg standing X-rays must be used to analyze the entire lower limb, identifying any contributions from distal segments [50]. Furthermore, the failure to consider all variables related to LLD and pelvic obliquity can significantly impact the evaluation of the harm as untreated or misassessed discrepancies can have repercussions on patients’ quality of life, including pain, functional disability, and gait disturbances.

A multidimensional approach, which accounts for all these aspects, is critical in establishing a correct causal relationship between the surgery performed and the reported damage. The medico-legal evaluation becomes even more complicated when comorbidities or individual conditions make the patient more vulnerable [51]. In such cases, the discrepancy must be considered a contributing factor to clinical deterioration. For example, a patient with pre-existing lower back pain may experience intensified discomfort due to LLD, but the underlying condition cannot be ignored.

The medico-legal significance of post-THA LLD largely depends on the quality of documentation provided by the surgeon and radiological assessment. Recent comparative studies have demonstrated the greater accuracy of 3D CT-based modeling over traditional 2D methods, although proper radiographic alignment is necessary to ensure precision [52]. In a medico-legal context, post-THA leg length discrepancy may be classified as iatrogenic if it is demonstrated that the surgical conduct did not adhere to the standards of care outlined in international and national guidelines. Apart from the lack of information about this complication, the failure to measure pre-operatively and plan the prosthesis, the absence of special precautions in patients with a higher pre-operative risk (significant preoperative limb length discrepancies, complex cases of congenital hip dysplasia, tissue laxity, etc.), and the early and unjustified indication for surgery as opposed to alternative conservative approaches could be assessed by the court-appointed technical experts as an aspect of deviation from the best practice required of the surgeon. Thus, recognizing a certain malpractice in the handling of the case that the judge could consider for the charge of liability.

After having ascertained the causation link between the LLD and the surgery, and the presence of improper pre- and/or intra-operative management, damage evaluation—ascertainment and quantification—arises.

The damage is defined as the permanent reduction of psycho-physical integrity in the form of permanent menomative and/or disabling sequelae, also called biological damage. This is comprehensive also of the relational and ergonomic aspects, expressed through limitations in social relationships and recreational opportunities. Sometimes a specific assessment of psychic permanent consequences may be useful, as it requires more specific methods of assessment and evaluation, so-called psychic damage, which is in any case counted in the all-inclusive assessment of biological damage.

Thus, determining the influence between signs and symptoms attributed to post-THA LLD and preexisting conditions often becomes central to the dispute in the damage evaluation. And, considering that the damage in functional LLD has a psychological component, preexisting psychological conditions and disorders have to be properly weighed.

The task of the medico-legal expert will be then to evaluate the damage attributable to improper or inadequate surgical performance [53]. It must also be determined whether the iatrogenic impairment interferes with the patient’s ability to perform work activities, accounting for residual pain-dysfunctionality, gait deficits, objective global static-dynamic postural alterations, and reduced tolerance for maintaining mandatory positions for prolonged periods. The patient’s perception of asymmetry may be exacerbated by post-operative anxiety and frustration, contributing to depression or failed surgery syndrome. This further damaging aspect belongs to the realm of psychological functioning and must be properly assessed in a medico-legal context. Damage evaluation must also address and weigh the use and effectiveness of correction devices (e.g., corrective insoles or the adoption of abnormal posture), and the involvement of other musculoskeletal structures compared to limb length discrepancy.

Usually, therefore, discrepancies of up to 10 mm, and in any case within 20 mm, are partially compensated for by corrections, thus providing a proportionate reduction in the damage evaluation. The greater the heterometry above this threshold, the greater the functional impairment and, therefore, the greater the valorisation, which in the case of very significant discrepancies (>50 mm) could be a kind of functional leg amputation. At these levels, which are more typically the result of major trauma surgery and therefore with less ability to control the discrepancy, the LLD is in fact assessed together with the broader spectrum of impairment with joint deficits and pain.The discrepancy becomes more significant in older subjects, as it may affect walking and, in some cases, may require the use of assistive devices, including wheelchairs, thus affecting personal autonomy, with subsequent damage quantification increase [44].

Future perspectives on LLD after total hip arthroplasty require further clinical investigation, particularly randomized controlled trials comparing different methods for assessing limb length. The goal is to identify the most reliable, cost-effective, and clinically applicable techniques that support performing the procedure in line with the standard of care.

From both clinical and medico-legal standpoints, a broader framework is needed to assess legal claims related to postoperative LLD, not only in terms of the numerical extent of the discrepancy but also considering its impact on the patient’s biomechanics and overall physiology. This would allow for more targeted, individualized, and meaningful evaluations.

## 7. Conclusions

The in-depth analysis of post-THA leg length discrepancy and its medico-legal implications has highlighted how proper management of limb length is a critical factor for the success of the procedure and the patient’s quality of life. Residual discrepancies following hip arthroplasty, although sometimes tolerable, can cause significant harm—not only in terms of physical pain but also from psychological and social perspectives—impacting mobility and participation in daily activities.

The surgeon must provide evidence of thorough medical history collection, comprehensive preoperative planning, and careful intraoperative evaluations (e.g., utilizing pelvic pins, calipers, or suturing tools). Pelvic obliquity and pre-existing comorbidities, also in the psychological area, must be considered preoperatively and intraoperatively. Those are also evaluated in case of litigation for LLD. Detailed documentation is essential not only for optimal patient management but also as evidence in the case of malpractice claims.

The quantification of damage related to post-THA LLD varies based on factors such as the extent of the discrepancy (e.g., <10 mm is tolerated by most patients), its impact on psycho-physical well-being and daily life activities, and the development of secondary pathologies, accounting for both pre-existing and post-operative factors.

## Data Availability

Data are not available.
